# Identification of genes associated with regenerative success of *Xenopus laevis *hindlimbs

**DOI:** 10.1186/1471-213X-8-66

**Published:** 2008-06-23

**Authors:** Esther J Pearl, Donna Barker, Robert C Day, Caroline W Beck

**Affiliations:** 1 Department of Zoology, University of Otago, P.O. Box 56, Dunedin 9054, New Zealand; 2 Laboratory of Molecular Organogenesis, Institut de Recherches Cliniques de Montreal (IRCM), 110 avenue des Pins Ouest, Montreal, QC H2W 1R7, Canada; 3 Biochemistry Department, University of Otago, P.O. Box 56, Dunedin 9054, New Zealand

## Abstract

**Background:**

Epimorphic regeneration is the process by which complete regeneration of a complex structure such as a limb occurs through production of a proliferating blastema. This type of regeneration is rare among vertebrates but does occur in the African clawed frog *Xenopus laevis*, traditionally a model organism for the study of early development. *Xenopus *tadpoles can regenerate their tails, limb buds and the lens of the eye, although the ability of the latter two organs to regenerate diminishes with advancing developmental stage. Using a heat shock inducible transgene that remains silent unless activated, we have established a stable line of transgenic *Xenopus *(strain *N1*) in which the BMP inhibitor Noggin can be over-expressed at any time during development. Activation of this transgene blocks regeneration of the tail and limb of *Xenopus *tadpoles.

**Results:**

In the current study, we have taken advantage of the *N1 *transgenic line to directly compare morphology and gene expression in same stage regenerating vs. BMP signalling deficient non-regenerating hindlimb buds. The wound epithelium of *N1 *transgenic hindlimb buds, which forms over the cut surface of the limb bud after amputation, does not transition normally into the distal thickened apical epithelial cap. Instead, a basement membrane and dermis form, indicative of mature skin. Furthermore, the underlying mesenchyme remains rounded and does not expand to form a cone shaped blastema, a normal feature of successful regeneration.

Using Affymetrix Gene Chip analysis, we have identified genes linked to regenerative success downstream of BMP signalling, including the BMP inhibitor Gremlin and the stress protein Hsp60 (*no blastema *in zebrafish). Gene Ontology analysis showed that genes involved in embryonic development and growth are significantly over-represented in regenerating early hindlimb buds and that successful regeneration in the *Xenopus *hindlimb correlates with the induction of stress response pathways.

**Conclusion:**

*N1 *transgenic hindlimbs, which do not regenerate, do not form an apical epithelial cap or cone shaped blastema following amputation. Comparison of gene expression in stage matched *N1 *vs. wild type hindlimb buds has revealed several new targets for regeneration research.

## Background

While all vertebrates are capable of some types of tissue regeneration, most, including humans, have lost the ability to regenerate whole structures such as limbs (*epimorphic regeneration*), [1]. Amphibians, in contrast, are exceptionally good at it: adult urodeles (newts and salamanders) and larval anurans (frogs and toads) can regenerate limbs, tails, jaws, and, in some cases, even the lens of the eye [2]. Epimorphic regeneration can be thought of as occurring in two phases: wound healing and cell proliferation. Regeneration-competent wound healing of amphibian appendages is generally rapid and involves covering the wound surface with a specialised epidermis lacking a basement membrane and dermis [3,4]. Once the wound is healed, the cells of the stump must mobilise under the wound epidermis and begin the process of replacing lost tissues, by forming a proliferating blastema. In urodeles, some of these cells may derive from de-differentiation of stump cells [5]. Reserve stem cells (muscle satellite cells) are also recruited in both urodeles and anurans [6,7].

Gene over-expression analyses in *Xenopus *limb and tail regeneration have indicated that successful regeneration requires the re-activation of developmental FGF or BMP signalling pathways [8-12]. More recently, evidence for the involvement of another developmental signalling pathway, the Wnt pathway, has been presented for *Xenopus*, axolotl, zebrafish and chicken [13-15]. The Wnt pathway is postulated to act upstream of FGFs [14].

We have developed a heat shock inducible transgenic line (*N1*) of *Xenopus *in which the BMP antagonist Noggin can be induced at a specific time during either development or regeneration, repressing BMP signalling [10,12]. We have used this line to show that BMP function is not only required for appendage regeneration but that it is specifically needed to generate a proliferating blastema while being dispensable for wound healing [12]. In *Xenopus*, limbs progressively lose the competence to regenerate as the tadpole undergoes metamorphosis [16,17] and cartilage becomes ossified [18]. Previous attempts to identify regeneration specific genes have compared tissues at very different stages of limb development, in which gene expression already differs [19-21]. In the current study, we have taken advantage of the *N1 *line to directly compare regenerating and BMP-signalling deficient non-regenerating tissue of the same developmental stage, in order to maximise identification of genes differentially regulated during the process of hindlimb regeneration.

Affymetrix GeneChip data was used to compare gene expression in stage 52 regenerating WT *Xenopus *limb buds and regeneration blocked transgenic *N1 *limb buds. Gene ontology (GO) analysis showed that genes involved in growth, development and the response to stress were statistically over-represented in regenerating WT hindlimb buds. GO categories relating to oxygen transport and epidermal development were over-represented in the non-regenerating *N1 *hindlimbs.

Several hundred genes were differentially expressed between WT and *N1 *hindlimbs 3 days after amputation. Differential expression was confirmed for 20 of these genes using quantitative RT-PCR. Further investigation by in situ hybridisation showed that the ability to upregulate and maintain expression of two of these genes, *Gremlin *and *Hsp60*, correlates with a successful regenerative outcome in *Xenopus *hindlimb regeneration.

## Results

### Comparison of N1 and WT limb buds following amputation

**Figure 1:**
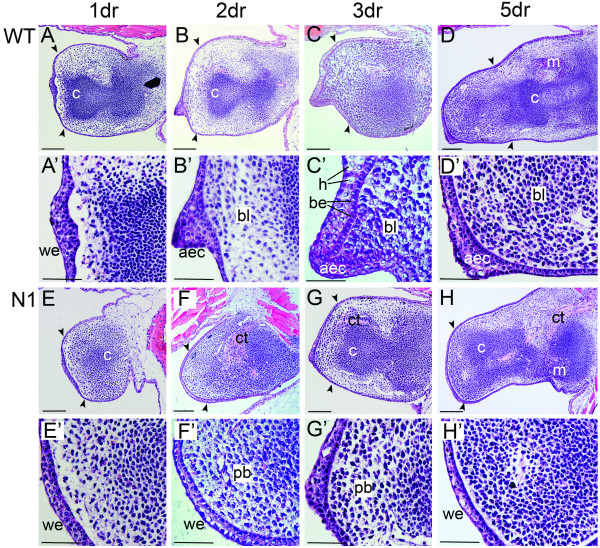
**Histological characterisation of hindlimb bud regeneration in WT and *N1 *tadpoles**. Representative haematoxylin and eosin stained 5 μm sections showing the phenotypic differences between regenerating WT and non-regenerating transgenic *N1 *hindlimb buds. Stage 52 limb buds were amputated at knee level and heat shocked as previously. Cartilage condensations are marked with a c. (A-D) WT limb buds, scale bars are 100 μm. Black arrowheads show the approximate plane of amputation. (A'-D') Higher magnification focusing on the distal area, scale bars are 50 μm. (A, A') wound healing has occurred after 24 hours and a wound epithelium (we) covers the cut site. (B, B') 2 days after amputation, blastema (bl) and AEC (aec) are forming. (C, C') 3 days after amputation, a cone-shaped blastema and the AEC are well established. Columnar basal epithelial cells (be) can be seen. Hypertrophic epithelial cells (h) can be seen in the AEC. (D, D') 5 days after amputation, the AEC and blastema are still apparent and outgrowth has begun. Muscle cells (m) can be seen proximally. (E-F) *N1 *limb buds, scale bars are 100 μm. Connective tissue (ct) is more obvious in these limb buds. E'-F' Higher magnification focusing on the distal area, scale bars are 50 μm. (E, E') wound healing has occurred after 24 hours, and a wound epithelium (we) covers the cut surface. (F, F') No AEC is apparent after 2 days and a rounded pseudoblastema (pb) forms proximal to the wound epithelium. (G, G') 3 days after amputation. (H, H') 5 days after amputation, the pseudoblastema has not expanded and a cell-free area of matrix is visible between the wound epithelium and underlying stump cells. Distal is to the left and posterior uppermost. dr = days of regeneration.

We have previously demonstrated that ectopic *Noggin *expression (and hence interruption of BMP signalling) from the *N1 *transgenic line causes hindlimb bud regeneration to fail at an early blastema stage resulting in the formation of a stump [12]. These results suggested that formation of the wound epithelium, which occurs in the first 24 hours following amputation, before this arrest, would be unaltered in *N1 *tadpoles. Analysis of these early stage regenerates by differential interference contrast (DIC) microscopy showed no obvious difference between the WT and *N1 *stumps. In both cases, a wound epithelium had formed over the surface by 24 hours and a blood clot could be seen underneath (data not shown). Sectioning revealed a wound epithelium approximately two cells thick covering the amputation plane and accumulation of mesenchyme underneath the wound epithelium (distal to the cartilage condensation) was present in both *N1 *and WT hindlimb buds, indicating the formation of an early stage blastema (Fig. 1A, A', E, E').

WT regenerating limb buds displayed a consistent phenotype at 2 days post amputation. A thickened apical epithelial cap (AEC), approximately five to six cells deep, had formed distally (Fig. 1B, B') replacing the thinner wound epithelium. The basal cells of the WT AEC had become noticeably columnar. These cells are believed to be important in signalling to the underlying mesenchyme, maintaining the blastema [18,22], therefore the presence of the columnar basal epithelial cells is indicative of regenerative potential. Accumulation of mesenchyme had resulted in formation of a cone shaped blastema, a characteristic also indicative of successful regeneration [18,23]. In contrast, the phenotype of the *N1 *line was more variable at 2 days post amputation. Approximately half the limb buds displayed little or no regenerative characteristics, including a rounded blastema (which we refer to herein as the *pseudoblastema*) and failure of the wound epithelium to develop into an AEC (Fig. 1F, F'). The remaining half possessed a somewhat thickened AEC and cone shaped blastema, generally of poorer quality than those seen in WT regenerates (data not shown). Columnar basal epithelial cells were not seen in any of the *N1 *limbs.

At 3 days post amputation, outgrowth of the WT limb was more apparent than at 2 days, due to further accumulation of the blastemal cells and thickening of the AEC (Fig. 1C, C'). The pseudoblastemas of the *N1 *hindlimbs had not undergone the same level of outgrowth, and the distal epithelium was generally thin and the basal epithelial cells disorganised rather than columnar (Fig. 1G, G'). This disorganised nature of the basal epithelial cells suggests that the *N1 *epithelium is impaired in, if not devoid of, signalling ability. The presence of connective tissue within the regenerating limb is also suggested to be inhibitory to the regeneration process [18]. Although eosin-stained connective tissue was observed in both the WT and *N1 *hindlimbs, it was seen more frequently and in larger amounts in the *N1s *(Fig. 1F, G, H). By 5 days post amputation the difference in regenerative ability between the *N1 *and WT hindlimbs was clear; the WT limbs had undergone a large amount of outgrowth, whilst the *N1 *limbs had taken on a characteristic stump form due to a lack of outgrowth (Fig. 1D, D', H, H'). We also observed hypertrophic (dying) cells in the cuboidal intermediate layers of the WT AEC as early as 3 days after partial hindlimb amputation suggesting that regression of the AEC is already beginning at this early stage.

### Global analysis of differential gene expression in regeneration competent and non-competent hindlimb buds

Our previous results with *N1 *transgenic tadpoles have shown that these animals are incapable of limb regeneration following amputation of stage 52/53 limb buds at the future knee level, if the *N1 *transgene is activated during the early stages of regeneration [12]. However, as shown above, we do observe the formation of a pseudoblastema in these animals, indicating that the earliest events of regeneration do occur but that the stump tissue subsequently fails to establish regrowth of the missing stylopod and autopod. In the current study we have made use of this *N1 *pseudoblastema to look for genes that are differentially expressed between regeneration competent and incompetent hindlimbs at the same developmental stage. This is important because as the limbs are still developing, comparison of naturally regeneration incompetent stages (Stage 57+) with early limb buds is likely to highlight genes involved in differentiation of the developing limb rather than those specifically recruited during and critical for regeneration.

**Figure 2:**
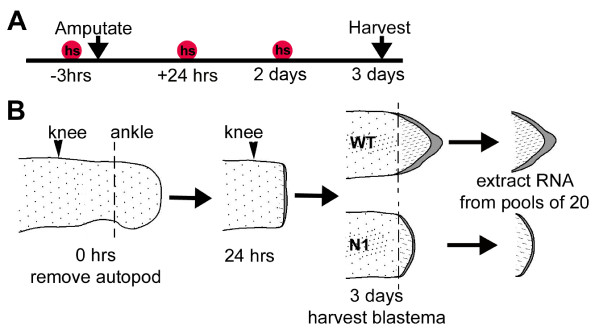
**Design of microarray experiment**. A) Timeline showing the treatments used to generate tissue for arrays. hs = heat shock, 30 minutes at 34°C. B) Stage 52 hindlimb buds were bilaterally amputated at the level of the future ankle (dotted line), defined by the anterior indentation, to remove the autopod. Knee level is marked by black arrowhead for orientation. Heat shocks were applied to both WT and *N1 *tadpoles as depicted in (A). After 3 days the blastemas were removed from the WT limbs and pseudoblastemas from the *N1 *limbs. BMP signalling is inhibited in the *N1 *limb buds, due to expression of *Noggin *from an inducible transgene, preventing successful regeneration. Pools of 20 blastemas or pseudoblastemas were used to extract RNA to generate microarray probes.

**Figure 3:**
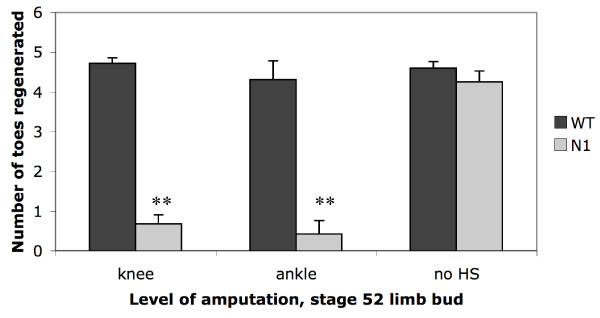
**Effect of *Noggin *over-expression on regeneration outcome following amputation at the future knee or ankle level**. Histogram of limb regeneration success as defined by the number of toes regenerated by stage 58 following amputation at either the future knee or ankle level of the limb bud at stage 52. WT and *N1 *animals were both subjected to heat shocks that activate transgene expression of *Noggin *in *N1*s. Non heat shocked controls were amputated at knee level. Error bars represent standard error and significant differences between WT and *N1 *animals are denoted by **).

Stage 52 blastema/pseudoblastema tissue for analysis was prepared as shown in Fig. 2. Two biological replicates were prepared from wild type (WT) and transgenic (*N1*) tissue following removal of the future autopod from both hindlimbs. This level of amputation was chosen so as to allow easier and more accurate removal of the blastema tissue. As the *N1 *transgene is activated by heat shock, all tadpoles including WTs were subjected to identical heat shocks 3 hours before amputation as well as 24 and 48 hours post-amputation (Fig. 2A). Blastema tissue from *N1 *pseudoblastemas or WT blastemas was collected 72 hours after amputation and RNA prepared from pools of 20 blastemas (Fig. 2B). Pools were used in order to generate enough RNA for unbiased amplification, according to published protocols [24]. Regenerative ability in *Xenopus *hindlimbs has been reported to degenerate in a proximal to distal direction [16,25,26], hence the possibility exists that regeneration from distal amputations could potentially be more difficult to inhibit effectively. Because previous results were obtained with limbs amputated more proximally, at the future knee level [12], a comparison of regenerative abilities was done using sibling tadpoles. In this case, only the right hindlimb bud was amputated at either the knee or ankle level at stage 52 (see Fig. 2) and the number of toes regenerated for each tadpole was determined at stage 58 (forelimb hatching). As expected, no significant difference in regenerative capacity was observed between ankle and knee level hindlimb amputations on WT animals (Fig. 3, Table 1). Furthermore, regeneration was significantly inhibited in heat shocked *N1 *tadpoles compared to equivalent WT siblings regardless of the level of amputation (Table 1, two sample t-test, *p *< 0.001). Healthy siblings produced from the same mating were used for all comparisons because variation in regenerative ability between different animals is widely acknowledged [27].

**Table 1:** Effect of *Noggin *over-expression on limb regeneration following amputation at the future knee or ankle level.

**Treatment**	**Type**	**Number of toes regenerated**						***N***	**Score/*N***	**% regenerated**	***t***	***p***
							
		**0**	**1**	**2**	**3**	**4**	**5**					
**Knee level amp.**	WT	0	0	0	1	4	10	15	4.6	100		
**no heat shock**	N1	0	0	2	2	2	10	16	4.3	100	1.08	0.293

**Knee level amp.**	WT	0	0	0	1	3	14	18	4.7	100		
**with heat shock**	N1	16	5	1	2	1	0	25	0.7	36	15.20	<0.001

**Ankle level amp.**	WT	1	1	0	0	0	11	13	4.3	92		
**with heat shock**	N1	10	1	0	0	1	0	12	0.4	16	6.71	<0.001

One limb bud was amputated (amp.) at the level of the future knee or ankle joint in anaesthetised tadpoles at NF stage 52. The contralateral limb was left intact. Where heat shock treatment is indicated, both WT and *N1 *tadpoles were heat shocked at -3 hrs, + 24 hrs and + 48 hrs relative to amputation time. The number of toes regenerated was recorded for each limb at stage 58. Two-sample t-tests were used to compare means, and significant difference was taken to be *p* > 0.05. There was no significant difference in regenerative ability between knee and ankle level amputations (*t *= 0.65, *p *= 0.524).

The four resulting RNA samples (2 biological replicates from *N1 *pseudoblastemas and 2 from WT sibling blastemas) were amplified using two rounds of in vitro transcription and used to probe Affymetrix *X. laevis *GeneChips. Following normalisation (MAS5 algorithm) the mean signals for *N1 *and WT replicates were calculated. WT biological replicates correlated well (Pearson coefficient = 0.98) whereas *N1 *replicates were slightly more variable (Pearson coefficient = 0.96). Fold changes were calculated from the mean signal for each probeset. 676 probesets were shown to be up-regulated >1.5 fold and 106 >2 fold in WT regenerating blastemas. A further 1170 probesets were >1.5 fold and 324 >2 fold up-regulated in *N1 *non-regenerating pseudoblastemas, in which BMP signalling is inhibited. An additional 165 probesets appeared to be only expressed in WTs while 83 were only expressed in *N1*s. Statistical analysis was performed using AffylmGUI [28].

### Validation of microarray results

The top 20 genes (based on largest fold change differential expression) up-regulated in either WT or *N1 *samples were re-annotated using Blast (NCBI) searches. The top 10 (WT) and top 11 (N1) genes that could be annotated were investigated further by qPCR. A further 2 genes that were expressed only in either WT (FGF-R3) or *N1 *(Keratin 18) were also investigated (Table 2). There was good agreement between qPCR calculated fold changes and those from the microarray data for 20 of these genes. Of the remaining 3 genes investigated, we were unable to generate reliable qPCR data for 2 and 1 was not confirmed as significantly differentially expressed.

**Table 2:** Selected differentially expressed genes between regenerating WT limb buds and non-regenerating transgenic *N1 *limb buds.

**Affymetrix Probeset(s)**	**Gene name**	**Array fold change**	***P*- value**	**qPCR fold change**	**Regeneration or developmental role**	**Pathway/function**

xl.22174.1.A1_at	*Hypothetical protein MGC68766*	3.85 WT	0.029	59.89	-	Homology to MHCII*α*

xl.23895.2.S1_at	Nucleoplasmin 3*	3.54 WT	0.003	4.04	-	Chaperone
xl.23895.1.A1_at		2.63 WT	0.011			

xl.24281.1.A1_at	*Hsp90*	3.13 WT	0.007	4.72	Muscle fibre regeneration [73]	Stress, chaperone

xl.16042.1.S1_at	*Type IX collagen*	2.94 WT	0.003	6.33	Bone formation and fracture [74]	Matrix, cartilage

xl.318.1.S1_at	*Gremlin*	2.85 WT	0.005	3.49	Limb development [31]	BMP inhibitor

xl.21917.1.S1_at	*Microtubule associated protein 1 light chain 3 α*	2.77 WT	0.003	1.52		Cytoskeletal protein

xl.6690.1.S1_at	*TIM22*	2.70 WT	0.008	1.92	-	Mitochondria transport

xl.17389.1.S1_at	*Tiarin*	2.70 WT	0.029	2.48	-	Early patterning

xl.8219.1.S1_at	Hsp60*	2.70 WT	0.286	2.62	Zebrafish *no blastema (nbl) *mutant [35]	Stress, chaperone
xl.8219.2.S1_at		1.81 WT	0.045			
xl.23194.1.S1_at		2.31 WT	0.049			
xl.24730.1.S1_at		2.11 WT	0.014			
xl.24730.1.S1_a_ at		1.94 WT	0.024			
xl.16470.1.A1_at	*3' exoribonuclease*	2.56 WT	0.040	3.66		RNA binding

xl.21891.1.S1_at	*Alcohol dehydrogenase 1*	9.7 *N1*	0.002	8.73	-	Retinoic acid synthesis

xl.7740.1.A1_at	*COX-2*	8.14 *N1*	0.048	5.54	Up-regulated in injured growth plate [75]	Inflammation

xl.9871.1.A1_s_a t	Thrombospondin 4*	7.32 *N1*	0.001	9.69	Expressed during osteogenesis [76]	Secreted, regulates cell interactions
xl.12952.1.S1_at		*N1* only	<0.001			

xl.11387.1.S1_at	*Haemoglobin α*	7.16 *N1*	0.004	6.23	-	Oxygen transport

xl.16451.1.A1_at	*Transmembrane serine protease 2*	6.96 *N1*	0.001	4.69	-	Protease

xl.9576.1.S1_at	*Carbonic anhydrase II*	6.52 *N1*	0.010	3.25		Metabolic enzyme

xl.8949.1.S1_at	*Ornithine decarboxylase 2*	4.48 *N1*	0.001	3.46	-	Metabolic enzyme

xl.2784.1.S1_at	Metallothionien A*	4.24 *N1*	0.023	4.20	Up-regulated in liver regeneration [77]. Up- regulated in skin wound healing [78]	Binds Zinc and Copper
xl.3144.1.S1_s_at		*N1* only	0.048			
xl.3144.1.S1_at		*N1* only	0.026			

xl.6874.1.S1_at	*Keratin 18*	*N1 *only	<0.001	4.31	-	Intermediate filament

xl.8908.1.S1_at	Raldh2*	3.01 *N1*	0.004	3.79	-	Retinoic acid synthesis
xl.18999.1.A1_at		*N1 *only	0.009			

Genes were selected from lists of up-regulated >2 fold in WT (regenerating, top section) or *N1 *(non-regenerating, lower section) and are listed in the order from the most differentially expressed according to Affymetrix GeneChip analysis (Array fold change, based on the mean of the two biological replicate samples). WT only or *N1 *only indicates that after normalisation of the array data, only WT or *N1 *samples respectively were called present by the GCOS software. *P *values were calculated for differential expression using AffylmGUI [28]. All the genes shown have been verified with qPCR and mean (from triplicate samples) normalised fold change values are shown (qPCR fold change). *denotes genes with 2 or more probesets on the chip, in each case all are up-regulated in the same direction. Where a known role in development or regeneration can be identified, representative references are given.

Fold change was calculated relative to change in expression of *FGF-10*, which was expressed uniformly across all microarray samples, and therefore used as a control gene for normalisation. In addition, the *X. laevis *GeneChip contains multiple probesets for some genes, and in every case where 2 or more probesets were identified for shortlisted genes, all were shown to be differentially expressed in the same direction, with the exception of Keratin 18 which was called absent in the duplicate probeset (Table 2). Several differentially expressed genes were identified that have known roles in regenerative processes (*COX-2, metallothionien A, Hsp90, Hsp60*), development (*Gremlin, Kruppel-like factor 2*, *Tiarin*) or osteogenesis (*Thrombospondin-4, Type IXα collagen*) as shown in Table 2 and references therein.

### Gene ontology

The microarray data was mined for Gene Ontologies to determine if any groups of genes, based on their predicted biological function, were consistently up-regulated. The *X. laevis *GeneChip is sparsely annotated, so Resourcerer [29] was used to obtain TC numbers and predicted gene ontologies. Statistically over-represented GOs were pulled from the 2 fold up-regulated shortlists for both regeneration competent WT hindlimb buds and the non-competent *N1*s (Table 3) by comparing gene numbers in the ontology group to the complete set of genes on the GeneChip. Duplicate probesets for the same gene were eliminated first to avoid skewing of the data. Genes involved in transport of proteins to the mitochondria, protein folding and re-folding and response to heat were all shown to be statistically over-represented in regenerating WT blastema and AEC (*p *< 0.01). These functional categories are all suggestive of a role for chaperones and cellular stress response in successful regeneration. Embryonic development, growth and positive regulation of growth rate categories were also over-represented (*p *< 0.01), as would be expected in regenerating tissue. In contrast, GO categories over-represented (*p *< 0.01) in the non-regenerating *N1 *pseudoblastemas included oxygen transport, cytoskeleton organisation and biogenesis, cell morphogenesis and adhesion, and epidermis development.

**Table 3:** Over-represented gene ontologies.

**GO Term**	**No. on array/total genes**	**No. >2x up-regulated**		***p***	**Gene ontology description**	**Contributing gene TC numbers**
					
		**Fraction**	**% genes on array**			
GO:0030150	5/13094	4/98	80%	1.47E-08	Protein import into mitochondrial matrix	TC261859, TC262099, TC275000, TC275032
GO:0006626	22/13094	5/98	23%	5.04E-07	Protein targeting to mitochondrion	TC261859, TC261956, TC262099, TC275000, TC275032
GO:0006457	82/13094	6/98	7%	3.33E-05	Protein folding	TC261741, TC261742, TC261767, TC262099, TC263531, TC275000
GO:0009408	27/13094	4/98	14%	4.54E-05	Response to heat	TC261767, TC262099, TC275000, TC275946
GO:0006458	2/13094	2/98	100%	5.54E-05	De novo protein folding	TC261859, TC275032
GO:0006628	2/13094	2/98	100%	5.54E-05	Mitochondrial translocation	TC261859, TC275032
GO:0042026	6/13094	2/98	33%	0.001	Protein refolding	TC262099, TC263531
GO:0040007	58/13094	4/98	7%	0.001	Growth	TC2612741, TC261742, TC262099, TC275000
GO:0006364	29/13094	3/98	10%	0.001	rRNA processing	TC261435, TC275446, TC285474
GO:0007098	8/13094	2/98	25%	0.002	Centrosome cycle	TC263531, TC274052
GO:0009792	96/13094	4/98	4%	0.006	Embryonic development (metazoa)	TC261741, TC261742, TC262099, TC288722
GO:0040010	60/13094	3/98	5%	0.010	Positive regulation of growth rate	TC261741, TC261742, TC288722

**GO:0015671**	**8/13094**	**5/295**	**63%**	**2.97E-07**	**Oxygen transport**	**TC262354, TC275583, TC275773, TC279855, TC289552**
**GO:0045104**	**10/13094**	**4/295**	**40%**	**4.76E-05**	**Intermediate filament organisation and biogenesis**	**TC260221, TC263972, TC286967, TC287917**
**GO:0030573**	**2/13094**	**2/295**	**100%**	**0.001**	**Bile acid catabolic process**	**TC264038, TC265106**
**GO:0015721**	**2/13094**	**2/295**	**100%**	**0.001**	**Bile acid transport**	**TC264038, TC265106**
**GO:0008544**	**38/13094**	**5/295**	**13%**	**0.002**	**Epidermis development**	**TC260221, TC263972, TC286967, TC287917, TC289357**
**GO:0000902**	**23/13094**	**4/295**	**17%**	**0.002**	**Cell morphogenesis**	**TC263972, TC286967, TC287917, TC289357**
**GO:0007155**	**109/13094**	**8/295**	**7%**	**0.003**	**Cell adhesion**	**TC260941, TC264675, TC274819, TC275243, TC275517, TC287476, TC288397, TC288694**
**GO:0006796**	**15/13094**	**3/295**	**20%**	**0.004**	**Phosphate metabolic process**	**TC265197, TC274479, TC292101**
**GO:0006810**	**234/13094**	**12/295**	**5%**	**0.007**	**Transport**	**TC262354, TC264038, TC264071, TC265834, TC271905, TC275007, TC275583, TC275773, TC276787, TC279855, TC288559, TC289552**
**GO:0006694**	**6/13094**	**2/295**	**33%**	**0.007**	**Steroid biosynthetic process**	**TC264038, TC265106**

Gene ontology terms (biological function) shown to be statistically over-represented (*p *< 0.01) in the WT (regenerating, top section) or the *N1 *(non-regenerating, bold, lower section) 2-fold up-regulated or more lists. TC numbers are tentative consensus numbers for the Affymetrix *X. laevis *GeneChip assigned by Resourcerer [29], around 1/3 of the genes on the chip were annotated using this method.

### Expression of *Gremlin* during limb development and regeneration

**Figure 4:**
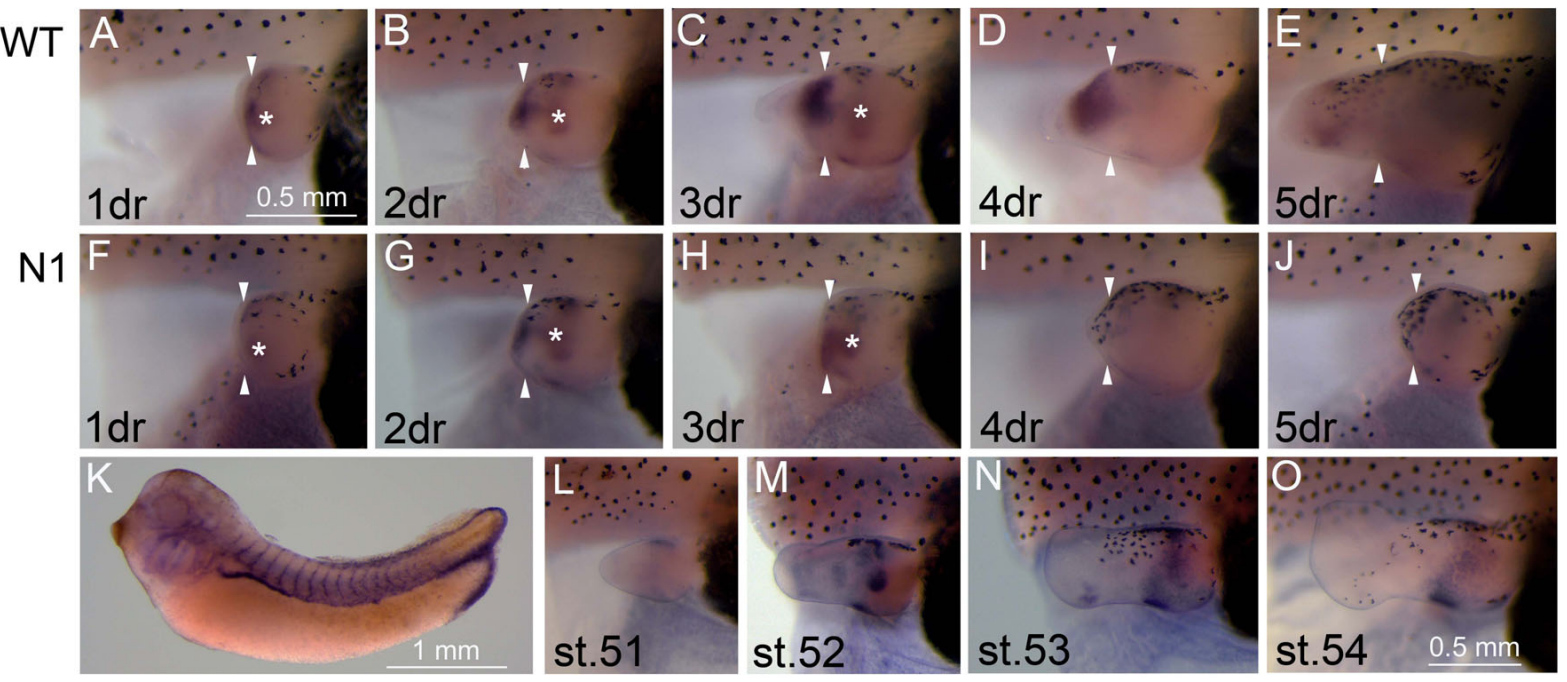
**Expression of *Gremlin *in regenerating WT and *N1 *limbs and during development**. Gene expression in regenerating wild type (WT) and *N1 *limbs and embryo tissue. (A-J) In situ hybridisation showing *Gremlin *expression in the regeneration bud. (L-O) Unoperated limb buds illustrating *Gremlin *expression during limb development. (K) In situ hybridisation showing *Gremlin *expression in a stage 32 embryo, the expression pattern is consistent with previously published *Gremlin *embryo expression [30]. White arrowheads indicate approximate amputation plane, scale bar in A applies to panels A-J and scale bar in O to panels L-O. In limb panels (A-J, L-O) posterior is uppermost, and distal to the left, dr = days of regeneration. In K, anterior is to the left and dorsal uppermost. White asterisks mark areas of *Gremlin *expression that are developmental and unrelated to regeneration.

One of the differentially expressed genes identified in our array screen was of immediate interest since it is known to be involved in both BMP signalling and limb development and patterning. *Gremlin *encodes a BMP inhibitor first isolated in *Xenopus *and expressed during development [30]. Subsequently, Gremlin has been shown to be involved in limb development in the mouse where it acts to regulate the signalling loop between Shh and FGFs in the posterior zone of polarising activity (ZPA), controlling the integrity of the AER [31]. Here, we show that *Gremlin *is also expressed in a specific pattern during *Xenopus *limb development (Fig. 4L–O) and regeneration (Fig. 4A–E). In tail bud stage embryos, *Gremlin *is expressed in the pronephros, pronephritic duct and neural crest cells of the head and trunk, as previously described [30]. During limb development, *Gremlin *is consistently expressed in a patch of anterior cells at a proximal-distal location corresponding to the future stylopod. There are also more dynamic areas of expression: early limb buds have *Gremlin *expressing cells in the posterior, paddle stage limb buds have more central areas of expression as well as two transient patches in the anterior of the forming footplate/autopod. These regions do not correspond to reported expression of *Gremlin *in the chick limb [32], which is incapable of regeneration.

During regeneration, *Gremlin *is expressed *de novo *by 24 hours after hindlimb amputation in the distal stump mesenchymal tissue but is absent from the epithelia and AEC (Fig. 4A). Expression appears to be restricted to the posterior half of the limb bud stump by 2 days post amputation and is absent from the distal blastema by 3 days (Fig. 4B, C). Expression appears to be down-regulated by 5 days (Fig. 4E) as the tissue begins to redifferentiate. *Gremlin *is also up-regulated following amputation of regeneration incompetent *N1 *hindlimbs but this appears to decline after 2 days so that expression is much reduced relative to WT controls by 3 days and is absent by 4 (compare Fig. 4D and 4I). The microarray data was generated using 3 day blastemas and pseudoblastemas, so the 3 fold upregulation in regenerating WT blastemas relative to pseudoblastemas corresponds to the expression shown in Fig. 4C and 4H. A clear difference is seen in all cases as shown by these representative limbs, and the decline of the pseudoblastema correlates with reduced *Gremlin *expression. Furthermore, *Gremlin *did not appear to be up-regulated in non-regenerating stage 57 *Xenopus *hindlimbs (data not shown). *Gremlin *upregulation appears to be specific to the limb regeneration process, as no expression was detected during tail regeneration (data not shown).

### Expression of *Hsp60* during limb regeneration

Analysis of the gene ontology showed that regenerating WT *Xenopus *hindlimbs significantly upregulate genes involved in protein folding and targeting to the mitochondrion. One of the genes with highest expression in WT blastema and AEC relative to *N1 *transgenic pseudoblastemas was *Hsp60*, (also known as GroEL) a chaperone involved in the folding and assembly of polypeptide chains into protein complexes (reviewed in [33]) and located primarily in the mitochondria [34]. Hsp60 already has a known role in vertebrate appendage regeneration: the zebrafish *no blastema *mutant (*nbl*) exhibits an early fin regeneration defect resulting from a loss of function mutation in the zebrafish homologue [35]. However, unlike Gremlin, Hsp60 has no reported role in limb development.

**Figure 5:**
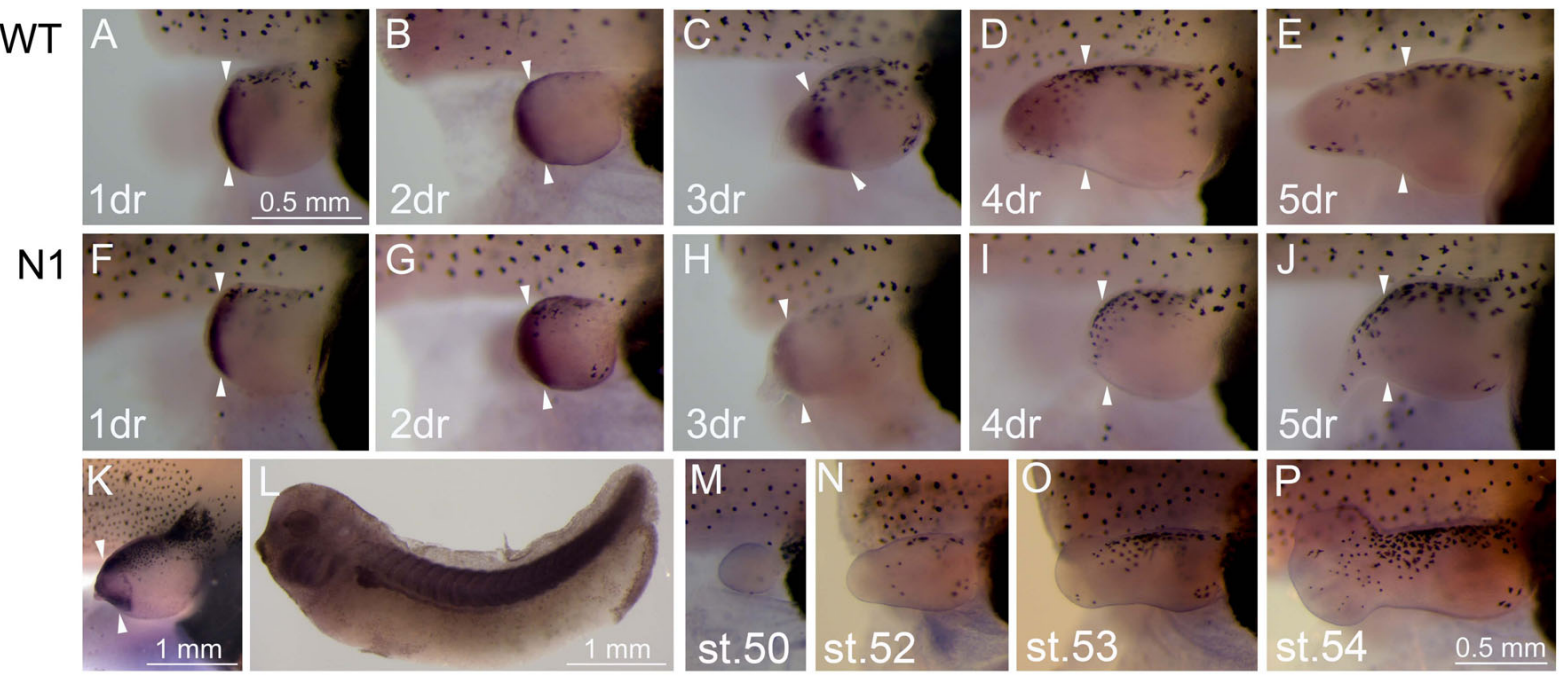
**Expression of *HSP60 *in regenerating WT and *N1 *limbs and during development**. Gene expression in regenerating WT and *N1 *limbs and embryo tissue. (A-J) In situ hybridisation showing *Hsp60 *expression in the regeneration bud. (M-P) Unoperated limb buds illustrating *Hsp60 *expression during limb development. (K) In situ hybridisation showing *Hsp60 *expression in stage 57 hindlimb of a WT animal 2 days after amputation. (L) In situ hybridisation showing *Hsp60 *expression in stage 32 embryo. White arrowheads indicate approximate amputation plane, scale bar in A applies to panels A-J and scale bar in P applies to panels M-P. In limb pictures (A-K, M-P) posterior is uppermost, and distal to the left, dr = days of regeneration. In L, anterior is to the left and dorsal uppermost.

We have looked at the expression of *Hsp60 *during limb development and regeneration. In tailbud stage embryos, *Hsp60 *is quite broadly expressed and there is especially strong staining in the pronephros, pronephritic duct and somites, eye and branchial arches (Fig. 5L). In limb bud stages, *Hsp60 *is notably absent from the hindlimb buds (Fig. 5M–P), suggesting that this gene is indeed not involved in limb morphogenesis. Strong expression in the distal mesenchyme/forming blastema is apparent 24 hours after amputation in both regeneration competent WT (Fig. 5A) and non-competent *N1 *hindlimb buds (Fig. 5F). This expression is maintained and somewhat expanded by 2 days after amputation, in a region corresponding to the expected location of the blastema of WT limbs and the pseudoblastema of *N1*s (Fig. 5B, G). By three days, however, a clear difference in expression is seen between *N1 *and WT hindlimbs, with expression maintained in the expanding WT blastemas but declining rapidly in the pseudoblastemas of the *N1 *hindlimb buds (Fig. 5C, H). After 4 days, *Hsp60 *expression is completely absent from the *N1 *pseudoblastema and is declining in the WTs, which are beginning to regenerate a new autopod and stylopod (Fig. 5D, I). By 5 days, *Hsp60 *expression is absent from the regenerating WT hindlimb buds (Fig. 5E). While expression of *Hsp60 *occurs in the early stages following amputation of either WT or *N1 *hindlimbs, possibly as a response to wound healing, only strong, maintained expression of *Hsp60 *in the blastema appears to be indicative of good regeneration.

In contrast to *Gremlin, Hsp60 *upregulation is not specific to limb blastemas. The gene is also re-expressed transiently in non-regenerating stage 57 limb buds, although in this case the expression appears to be localised to the anterior and posterior underlying mesenchyme (Fig. 5K). Expression is also up-regulated in the tail blastemas of non-regenerating refractory stage 47 WT tadpoles, and in regenerating stage 50 tadpoles, 2 days after amputation of the posterior half of the tail (data not shown).

## Discussion

### BMP signalling is required for transition of wound epithelium to the apical epithelial cap signalling centre in *Xenopus*

Our previous results have shown that the effect of inhibiting BMP signalling with ectopic *Noggin *under the control of the inducible *Hsp70 *promoter blocks regeneration most efficiently when targeted to the post-wound healing stage of regeneration (>24 hours post amputation). Histological analysis of *N1 *hindlimbs following amputation demonstrated that the AEC either fails to develop from the wound epithelium or is poorly formed and organised. In particular, the basal epithelial cells, which take on a characteristic columnar morphology during normal hindlimb regeneration, fail to do so in *N1*s, suggesting that BMP signalling is necessary to establish the normal morphology of the AEC. As these basal epithelial cells are thought to represent the AEC compartment responsible for signalling to underlying mesenchymal cells of the forming blastema in urodele amphibians [22], this signalling is likely disrupted or absent in *N1s*. In agreement with this previous study of axolotl limb regeneration, we can distinguish clearly between the early, 3 cell layer thick wound epithelium and the later developing multilayered AEC of *Xenopus*. These authors further suggest that the cuboidal basal epithelial cells of the AEC functionally mimic the apical ectodermal ridge (AER), which forms the signalling centre driving outgrowth and patterning of the developing limb [22]. If the same is true in *Xenopus*, then the failure of this compartment to develop may explain the degeneration of the pseudoblastema and ultimate failure of *N1 *hindlimbs to regenerate. Analysis of the microarray data revealed that genes related to epidermis development are over-represented in the *N1 *amputated hindlimb samples, suggesting that by 3 days post amputation the *N1 *line is producing mature skin over the wound.

BMPs have multiple roles during limb development in vertebrates [36,37]. BMP signalling plays a role in AER formation, patterning, growth and regression [38-43] as well as later roles in differentiation of the cartilage and bone and sculpting of the digits by apoptosis [42,44-48]. Much evidence has accumulated that demonstrates that these multiple roles of BMPs during limb development are tightly regulated by a number of secreted antagonists expressed in the forming limbs [46,49,50].

Our results suggest that BMPs act to regulate AEC induction during regeneration of the hindlimb bud, as the loss of BMP signalling in the *N1 *line, depending on the individual tadpole, results in either the absence or impaired formation of the AEC. Loss or impairment of the AEC would be expected to ablate or decrease the level of signalling to the underlying mesenchyme resulting in decreased proliferation of the *N1 *mesenchyme. Previously, we have shown that proliferation is indeed decreased in the *N1 *hindlimb pseudoblastema relative to the regenerating WT blastema [12]. Partial regeneration (reduced patterning) may arise as a result of reduced AEC function, leading to the variability seen in regenerative success.

### The stress response is specifically activated during regeneration

A comparative analysis of global gene expression in 3 day post-amputation regenerating (WT) versus non-regenerating (*N1*) hindlimb buds was performed. Genes differentially expressed by more than 2-fold between WT and *N1*s were searched for significantly over-represented Gene Ontologies (GO: biological function). Of the 12 GO terms significantly over-represented in WT, regenerating limb buds, 7 are linked to chaperone activity, suggesting that appropriate stress response processes correlate with regenerative success. Two genes in this group, *Hsp60 *and *Hsp90*, were confirmed as over-expressed in WT regenerates using q-PCR. *Hsp60 *was subsequently demonstrated to be induced in the distal mesenchyme following hindlimb bud amputation, and maintained only in successful regenerates. Without BMP signalling, the expression of *Hsp60 *declines much sooner, and the pseudoblastema regresses. A previous study showed that *nbl (no blastema) *zebrafish mutants fail to regenerate their fins following partial amputation because of a missense mutation in Hsp60 which alters the ability of the protein to bind to and fold or re-fold denatured proteins [35]. Zebrafish fin regeneration goes through the same processes of wound epidermis formation and blastema formation that drives regeneration in tetrapod limbs [51]. Another member of this family, Hsp70, is expressed during development and regeneration in the axolotl limb [52]. In urodele amphibians such as the axolotl, there is thought to be a de-differentiation step, which occurs following wound healing and provides cells to the blastema. *Hsp70 *expression was strong and maintained in the blastema following axolotl forelimb amputation, similar to the expression of *Hsp60 *presented here. Therefore the urodele and anuran amphibians, which utilize different mechanisms for forming the blastema, both appear to require induction and maintenance of heat shock proteins. Hsp70 has also been linked to arm regeneration in echinoderms [53] suggesting that this is not unique to vertebrate regeneration.

The partial loss of a limb or fin likely subjects the remaining cells to a number of stresses. The heat shock proteins (Hsps) were initially discovered by virtue of their induction by elevated temperature in the fruit fly *Drosophila melanogaster *[54]. However, they are now known to have a more general role in cellular stress and are even released from cells under some circumstances, where they modulate inflammatory and immune responses (reviewed in [55]). We do not currently know whether the Hsps induced in regenerating limbs are released into the extracellular spaces, but this provides an attractive mechanism for generating a regeneration niche. Certainly, *Hsp60 *induction seems to be a requirement for regeneration of diverse tissues, including the zebrafish fin and heart [35], and frog limb bud and tail. Interestingly, we are utilizing the mechanism of heat shock to induce ectopic *Noggin *expression in order to block BMP signalling in our *N1 *transgenic line. Hence, we would expect that heat shock proteins in general may be elevated in both transgenic and WT animals, since both are subjected to the same heat shock protocol. We have observed a general, ubiquitous increase in *Hsp60 *transcripts in WT animals that have been subjected to heat shock, which does not seem to be apparent in *N1 *siblings (data not shown). This may suggest that BMP signalling activity in fact modulates the induction of Hsp60 and that this prevents *N1 *tadpoles from being able to regenerate efficiently.

### A possible role for the BMP inhibitor Gremlin in limb regeneration

Comparison of gene expression in regenerating and BMP signalling deficient, non-regenerating limb buds using a same stage microarray approach identified the developmentally important gene *Gremlin *as up-regulated during regeneration. Further investigation revealed that *Gremlin *is initially up-regulated in distal mesenchyme regardless of regenerative potential, but that it is rapidly lost by hindlimbs that subsequently fail to regenerate. *Gremlin *is involved in limb development in chickens, and knockout mice show defects in limb outgrowth and patterning due to a loss of reciprocal signalling between the AER and underlying ZPA, located posteriorly [31,32]. Here, we show that *Gremlin *is also expressed during limb development in *Xenopus *although the pattern of expression does not appear to mimic that seen in chick and mouse, suggesting that it may play a different role in non-amniote limb development. During regeneration however, the highest expression of *Gremlin *is seen in the posterior half of the mesenchyme underlying the wound epithelium. As the AER develops in WT regenerates, this expression first intensifies from 2–3 days post amputation and then gradually declines, becoming absent by 5 days. It is interesting that this expression pattern does not recapitulate developmental *Gremlin *expression in *Xenopus*, rather resembling the expression seen in developing amniote limbs.

Gremlin, like Noggin, is a BMP antagonist, but it also down-regulated by *Noggin *[56,57], which may account for the rapid loss of induced *Gremlin *in the *N1 *hindlimb buds. Interestingly, the loss of *Gremlin *in failed regeneration occurs between 2–4 days post amputation, when the pseudoblastema is regressing. Furthermore, the upregulation of *Gremlin *does not occur following tail amputation or removal of late stage hindlimbs that are unable to regenerate efficiently, suggesting that the ability to upregulate and maintain *Gremlin *expression in the mesenchyme could be important for limb regeneration.

### New target genes for regeneration research

Two previously published studies have used differential screening to identify genes associated with regenerative success in *Xenopus *tadpole hindlimbs [19,21]. Comparison of our data with these previous studies does not indicate a high level of gene discovery overlap. However, this is unsurprising for two reasons. Firstly, both previous studies compared markedly different stage limbs: stage 53 (regenerative) and stage 57 or 59 (non-regenerative). This means that limb tissue of very different maturity and differentiation status was being compared, perhaps leading to identification of genes involved in this maturation process as well as regeneration specific genes. Secondly, the tissue was collected after either 1 or 5 days of regeneration in Grow et al [21] and after 7 days in King et al [19]. The current study focused on comparisons at 3 days after amputation, where the blastema and AEC are well established, but before differentiation begins. Neither of the two genes studied in detail here would have been likely to show differential expression at either 1, 5 or 7 days post amputation, and therefore were likely to be missed by previous investigations. Furthermore, our current study is likely to favour the identification of genes which act downstream of BMP signalling during limb regeneration.

## Conclusion

*N1 *transgenic tadpoles, which are deficient in BMP signalling, probably fail to regenerate because they do not form a morphological AEC. While mesenchyme accumulates beneath the wound epithelium in these animals it forms a rounded, regeneration incompetent pseudoblastema rather than the cone shaped, regeneration competent blastema seen in WTs. By comparing gene expression in same stage *N1 *and WT hindlimbs 3 days after amputation, we have identified several genes and functional groupings associated with regenerative success in *Xenopus *tadpole hindlimbs. Further investigation of these may reveal new potential therapeutic targets for regeneration research.

## Methods

### Transgenic animals

The *N1 *stable line of transgenic *Xenopus *has been previously described [12]. Briefly, the animals contain a transgene comprised of two linked parts, the first containing *X. laevis *Noggin coding sequence [58] under the control of the *Hsp70 *promoter, and the second the green fluorescent protein (GFP) coding sequence under the control of the lens specific promoter *γ-crystallin*. The line is derived from a single insertion founder made by sperm nuclear injection using the method of Kroll and Amaya [59] modified as in Beck et al [10]. All animal experiments were subject to New Zealand's animal welfare standards for vertebrates and were reviewed by the University of Otago Animal Ethics Committee (AEC). The AEC approved all experiments under protocols AEC57/03 and AEC78/06.

### Microarray analysis

WT and *N1 *strain tadpoles were grown to stage 52 [60] and heat shocked at 34°C for 30 minutes to activate the transgene. After 2–3 hours, hindlimb tissue was removed at the future ankle using Vannas iridectomy scissors. Tadpoles were heat shocked again 24 and 48 hours post-amputation to maintain transgene expression. Three day post-amputation blastema or pseudoblastema tissue was isolated as shown in Fig. 2 from 2 × 20 WT and 2 × 20 *N1 *blastemas and stored in RNA *later *(Qiagen) at 4°C until extraction. Total RNA from each biological replicate was subsequently isolated using TRIzol (Invitrogen) and cleaned using an RNeasy mini kit (Qiagen). Half of the total RNA from the four samples was sent to the Centre for Genomics and Proteomics at the University of Auckland, for labelling and hybridisation to *Xenopus laevis *GeneChips (Affymetrix), enabling analysis of over 14,400 transcripts. RNA was assessed using capillary electrophoresis (Bioanalyser, Agilent). Labelling of each sample was performed using the Affymetrix 2-cycle kit. Microarray data was normalised in Affymetrix GeneChip Operating Software (GCOS) using the MAS5 algorithm [61] and fold change calculated for each probeset by comparing the mean value for the 2 *N1 *samples to the mean value for the 2 WT samples. Statistical analysis was conducted using the Bioconductor software AffylmGUI [28], which analyses differential expression in terms of linear models and generates a moderated t-statistic and *P *value for each probeset. The data discussed in this publication have been deposited in NCBIs Gene Expression Omnibus (GEO) [62] and are accessible through GEO Series accession number GSE9813.

### Gene ontologies

*Xenopus *TC (Tentative Consensus) numbers and Gene Ontology (GO) assignments for biological function were obtained for the Affymetrix *Xenopus laevis *GeneChip using Resourcerer v13.0 [29], December 2006 release [63]. 2-fold or greater up-regulated lists of TC numbers were created for both WT and *N1 *blastemas and duplicate TC numbers (arising when the GeneChip contained multiple probe sets for one gene) removed using the online BAR duplicate remover tool [64]. Genemerge v1.2 [65] was used to determine GO groups which were statistically over-represented in the WT or *N1 *2-fold up-regulated lists compared to the genes represented on the array.

### Quantitative real time PCR (qPCR)

In preparation for qPCR, mRNA was amplified for one cycle from the remaining RNA using a MessageAmp™ II aRNA Amplification kit (Ambion). Reverse transcription was performed using 500 ng amplified RNA per reaction, using random primers (Invitrogen) and Superscript™ III (Invitrogen). The resulting cDNA was diluted 1:100 for use in qPCR reactions.

qPCR was performed on a Stratagene Mx3000P system using ABsolute QRT-PCR SYBR Green Mix (ABgene), 140 nM forward and reverse primers and 9 μL diluted cDNA. For each sample, qPCR reactions were prepared in triplicate and compared to a single no reverse transcriptase (RT) control to check for genomic contamination. A standard 40-cycle program with hot start was used and annealing temperatures varied from 55–62°C. Melting curves were examined to confirm specificity of product amplification. The NCBI program Spidey [66] was used to predict intron-exon boundaries by comparing *X. laevis *cDNA sequence to *X. tropicalis *genomic and transcript sequence from the Joint Genome Institute [67]. Primers were designed in Oligoperfect (Invitrogen) so that one of the primers spans two predicted exons. Primers used, annealing temperatures and product sizes are in Additional file 1.

*FGF-10 *expression levels did not change between *N1 *and WT samples and was therefore used to normalise data from qPCR before calculating the fold change from mean ΔCT values. Estimates of primer efficiencies were first calculated using LinRegPCR [68]. These estimates of primer efficiency (and the standard error associated, calculated using Microsoft Excel) were then entered into REST, an Excel-based macro that calculates fold changes with and without normalisation to a reference gene [69,70]

### In situ hybridisation

The full length coding sequence of *Gremlin *was amplified from stage 12 *X. laevis *embryos by RT-PCR using High Fidelity Platinum *Taq *(Invitrogen) and ligated using flanking *XbaI *and *KpnI *sites into *pBluescriptIIKS+ *(Stratagene). Oligonucleotide primers used were 5' gctctagaatgaactgtctcgtttatgc, 3' gcggtaccttagtccaggtctatgg. The full length coding sequence of *Hsp60 *was amplified from stage 17/18 *X. laevis *embryos by RT-PCR using High Fidelity Platinum *Taq *(Invitrogen) and ligated using flanking *XbaI *and *KpnI *sites into *pBluescriptIIKS+ *(Stratagene). Oligonucleotide primers used were 5' cgctctagaatgctgcggctac, 3' gcggtaccttaacaaagcaacttacc. Insertions were verified by DNA sequencing, performed at the Allan Wilson Centre for Genome Service (Massey University, New Zealand). Digoxygenin labelled ribonucleotide probes were made by linearising plasmids with *XbaI *and transcribing using T3 polymerase labelled with digoxigenin-UTP labelling mix (Roche). DNase I (Invitrogen) was used to remove templates following transcription and the probes were precipitated with 2.5 M LiCl. Whole-mount in situ hybridisation of embryos and tadpoles was performed as previously described in [71] with modifications as in [72].

### Histology

Tadpoles were fixed overnight in cold ethanol/glycine fixative (70% ethanol, 15 mM glycine pH 2.0) at -20°C, dehydrated in methanol and embedded in paraffin wax. 5 μm sections were cut using a Leica microtome and stained with haematoxylin and eosin.

## Authors' contributions

CWB conceived the work and supervised it. RCD performed analysis of the microarray data. EJP, DB and CWB performed the experiments. CWB wrote the manuscript with input from all authors.

## Supplementary Material

Additional file 1 Supplementary table S1. Contains primer sequences used for qPCR. Click here for file
