# Supplementary material for: Identification of genes associated with regenerative success of Xenopus laevis hindlimbs
Source: BMC Dev Biol. 2008 Jun 23;8:66. doi: 10.1186/1471-213X-8-66 (PMC2483965; doi:10.1186/1471-213X-8-66)
Supplement: Additional file 1 — Supplementary table S1. Contains primer sequences used for qPCR. [file 1471-213X-8-66-S1.doc]

| **Gene name** | **Genbank accession number** | **5’ primer** | **3’ primer** | **Annealing temp** | **Product**  **size** |
| --- | --- | --- | --- | --- | --- |
| *Gremlin* | AF045798 | ccttcccacaggatgaactg | ggctccctgtgatccactaa | 55°C | 114bp |
| *Thrombospondin 4* | BC077261 | catgcgtggaggaagactg | cctctgggattgtatcattacacc | 55°C | 95bp |
| *Haemoglobin α* | BC073064 | ggaaactttgggctgctgt | caaggaacttgtcccaagca | 60°C | 103bp |
| *Hsp60* | BC046687 | gcgttgcagtactaaaggttg | tccttcttccacagctgcac | 55°C | 107bp |
| *TIM22* | BC084949 | ccgactgctaaagaggtgct | tcctcgatatgattccaccag | 60°C | 120bp |
| *Hsp90* | BC072998 | gacttggtcttggaattgatga | tccattcgagaagagtctccat | 55°C | 109bp |
| *Tiarin* | AB075925 | cacaaccttctattagttatggaactg | ccttaccccatcctccaaat | 60°C | 120bp |
| COX-2 | BC077809 | agaccagcagggttcaaact | gttgaggcagcacaggacat | 60°C | 120bp |
| *Transmembrane serine protease 2* | BC084671 | tcctttgggtatggacagtg. | agctgccagacctggagtaa | 55°C | 95bp |
| *Ornithine decarboxylase2* | AF217544 | ggattacagcaaaccgagga | gtggctgcaaaggtacgagt | 60°C | 101bp |
| *MHCIIα-like* | BC060437 | ttcacaaattcccaattcca | gctttaggcaatcaagaaaaaca | 55°C | 101bp |
| *Nucleoplasmin 3* | BC078496 | ccatgtgctctacggtagtcg | aaagctggtgttggaatcct | 55°C | 102bp |
| *Type IX collagen* | BC045244 | tctgtaaatgtgttatgtgggtca | caatgcaatcttaggccgata | 55°C | 101bp |
| *Metallothionein A* | BC091777 | aaaagctgctgttcctgctg | gacgcagagcccttcagtta | 55°C | 109bp |
| *Raldh2* | AF310252 | gcaggaactgtttggataaattg | ctctcccatttctcttccattg | 62°C | 96bp |
| *Microtubule associated protein light chain 3α* | BC043946 | ggcaattccgttgataggg | tttctgttccgaccctccac | 55°C | 95bp |
| *3’exoribonuclease* | BJ077527 | tccatgtagagtcatttctaaacca | tcttctgattcgtcaatttgg | 60°C | 102bp |
| *Alcohol dehydrogenase 1* | BC041319 | ccaaagtagaccctgctgct | tggagtaaccttagcagtgttca | 55°C | 98bp |
| *Keratin 18* | BC054993 | tcaaaggtggaggagcacac | taagctggctttctggttgc | 60°C | 144bp |
| *Carbonic anhydrase II* | CB560639 | acgtgatgtttttgctctcg | cgcaagtggaacttttacgg | 55°C | 102bp |
| *FGF-10* | AB73747 | cagtgaaagccattaacagca | ggtgttgtagccattctcctct | 55°C | 125bp |
